# The impact of filamentous plant pathogens on the host microbiota

**DOI:** 10.1186/s12915-024-01965-3

**Published:** 2024-08-15

**Authors:** Victor M. Flores-Nunez, Eva H. Stukenbrock

**Affiliations:** 1 Environmental Genomics, Christian-Albrechts University, 24118 Kiel, Germany; 2 Max Planck Fellow Group Environmental Genomics, Max Planck Institute for Evolutionary Biology, 24306 Plön, Germany

**Keywords:** Plant microbiome, Fungal plant pathogens, Pathogen effectors, Microbial interactions

## Abstract

When a pathogen invades a plant, it encounters a diverse microbiota with some members contributing to the health and growth of the plant host. So far, the relevance of interactions between pathogens and the plant microbiota are poorly understood; however, new lines of evidence suggest that pathogens play an important role in shaping the microbiome of their host during invasion. This review aims to summarize recent findings that document changes in microbial community composition during the invasion of filamentous pathogens in plant tissues. We explore the known mechanisms of interaction between plant pathogens and the host microbiota that underlie these changes, particularly the pathogen-encoded traits that are produced to target specific microbes. Moreover, we discuss the limitations of current strategies and shed light on new perspectives to study the complex interaction networks between filamentous pathogens and the plant microbiome.

**Supplementary Information:**

The online version contains supplementary material available at 10.1186/s12915-024-01965-3.

## The plant microbiome is important for plant health and defense

The plant microbiome is undeniably relevant for the development and health of the host. The microbiome of plants comprises a diverse and numerous array of microorganisms (viruses, bacteria, archaea, fungi, and other microeukaryotes) that colonize the inside and outside of plant tissues, in underground and aboveground organs [1]. The plant microbiome contributes collectively to the host’s fitness since “microbe-free” plants or those with reduced microbiota develop poorly compared to plants associated with more complex microbial communities [2, 3]. The role of the plant microbiome has been successfully assessed in experiments with the plant model *Arabidopsis thaliana*, which can be propagated in sterile conditions and inoculated with synthetic microbial communities. This strategy has allowed the dissection of mechanisms that govern the assembly and function of plant microbiomes [4].

Amplicon sequencing from plant metagenomic DNA has uncovered the vast diversity of host-associated microorganisms and the biotic and abiotic factors that correlate with microbial composition. This approach relies on the amplification and sequencing of the ribosomal markers 16S rRNA and ITS region to determine the composition of the prokaryotic (bacteria, archaea) and eukaryotic (fungi and protists) microbial communities, respectively. With this type of amplicon data, the differences in the microbiome composition can be evaluated based on the alpha diversity (richness and evenness) and abundance of amplicon variants (operational taxonomic unit (OTU), amplicon sequence variant (ASV), etc.) as well as on the beta diversity (reflecting dissimilarity in the composition of amplicon variants). Network analyses of amplicon data can moreover be used to infer patterns of interactions among microbiota members.

Comparative studies of microbiomes have documented that different plant species (including domesticated and non-domesticated species) have different microbiome compositions [5, 6]. Also, the plant microbiome can vary among genotypes pertaining to the same species [7, 8] suggesting a significant impact of genetically encoded traits and their response to the other biotic and abiotic factors. The biotic factors include the developmental stage of the plant, the invasion of pathogens and herbivory, and, importantly, the native microbial communities of the soil [9]. Abiotic factors, which also influence microbiome composition, include the physicochemical properties of the soil and the environmental climate factors such as temperature, rain, and pollutants [9]. Consequently, the plant microbiome should be considered as a dynamic feature, whereby microbial community assembly is determined by the interaction of multiple factors that vary in time and space. Despite these variations, accumulating evidence has demonstrated the presence of a “core” microbiome that is consistently present and shared among different plant hosts and conditions. Such “core microbiomes” have been the focus of many studies as these are considered ecologically and functionally important to the host [10].

Much attention has been dedicated to identifying the underlying mechanisms of microbial growth promotion and stress resistance [1]. Bacteria and fungi can directly promote the growth of plants by facilitating the availability and uptake of nutrients [11]. Moreover, the mitigation of abiotic stress can be associated with the microbial production of growth regulators and detoxifying enzymes by members of the plant microbiome [12, 13]. Importantly, microbiome screenings have also demonstrated that plants contain microorganisms capable of inhibiting plant pathogens or reducing the severity of pathogen infection [14].

Plant pathogens are microorganisms (such as virus, bacteria, fungi, etc.) that cause damage to their plant hosts by inducing disease. Plant diseases and pests are a threat to food production and security as they, on average, account for 40% of crop losses [15]. Filamentous pathogens such as fungi and oomycetes include some of the most devastating plant pathogens. These pathogens are unique in terms of their infection mechanisms and lifestyles [16]. Broadly, they are classified as biotrophic when they colonize and thrive in living tissue and necrotrophic when they induce cell dead to get nutrients or both [17]. Plant immune responses to these two groups of pathogens differ considerably, but both involve hormonal signaling and transcriptional activation of specific and unspecific antimicrobial genes. Moreover, also, the plant microbiota responds to the invasive growth of pathogens in the host tissue.

The plant microbiota have different mechanisms to antagonize filamentous pathogens, for example, the production of antifungal metabolites, enzymes that degrade the fungal cell wall, the competition for nutrients and space, and mycophagy, among others [18, 19]. While the field of biocontrol research has long been studying suppressive microorganisms with the aim of producing commercial inoculants to control plant diseases, pioneer research showed that fungal pathogens also antagonize the resident microbiota and modify the host microbiome to stimulate disease progression [20].

**Fig. 1:**
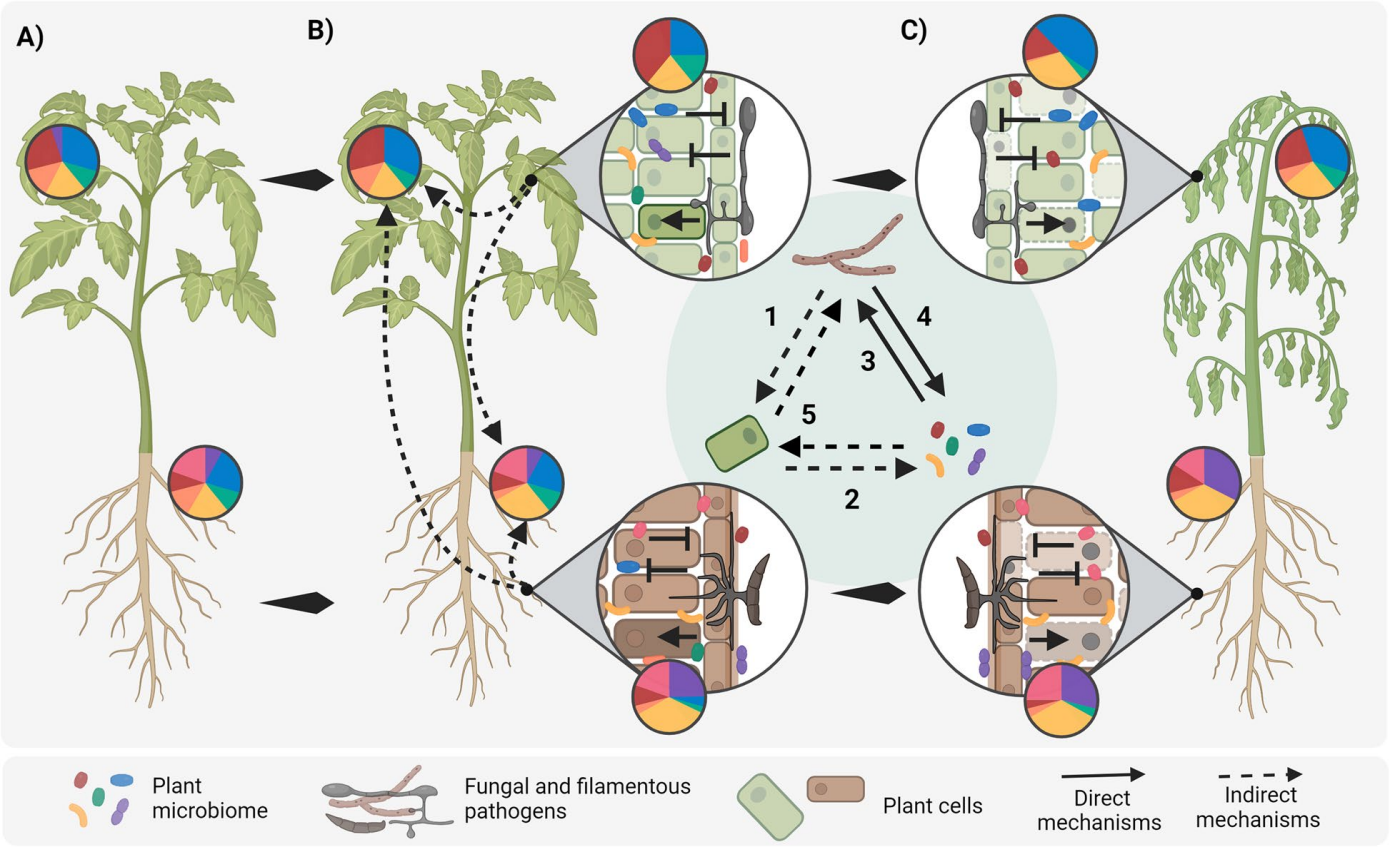
Filamentous plant pathogens induce changes in the host microbiome. A plant harboring a defined microbiome (A) is infected by leaf or root filamentous pathogens. Plant pathogen infection influences the microbiome composition locally and systemically (B). Diseased plants harbor a microbiome different from healthy plants (C). Changes in the microbiome composition arise from the interaction between the pathogen, host, and microbiome (inner circle). The plant pathogen triggers immune responses and an alteration of plant metabolism (1) that indirectly influences the plant microbiome (2). The plant microbiome harbors microorganisms that directly compete against pathogens and contribute to disease suppression (3). Filamentous pathogens can antagonize the host microbiome to modify the microbial niche and stimulate disease progression (4). The microbiome can indirectly influence disease progression, for example by inducing systemic resistance (5, not reviewed here)

The plant microbiome and plant pathogens coexist and interact in the host during disease progression. The microbiome that is associated with a host in the event of pathogen invasion and disease is defined as a “pathobiome” [21]. When microbiome disbalances result in damage to the host, it is considered a state of “dysbiosis,” for example, a deficiency in the host’s immune signaling pathways that allows ordinarily commensal microorganisms to cause damage [22]. The plant pathobiome arises from the interaction between the host, pathogen, and microbiome (Fig. 1). Notably, fungal plant pathogens manipulate the plant’s immune response and metabolism via the secretion of apoplastic and cytoplasmic effectors [23], which, indirectly, may lead to a change in microbiome composition. Moreover, pathogens have also evolved strategies to interact directly with the plant-associated microbiota, for example via the secretion of antimicrobial effectors [20]. Thereby, the influence of plant pathogens on the microbiota composition of the host can arise from both indirect mechanisms through the host and direct microbial interactions (Fig. 1).

To better understand how filamentous pathogens interact with the host microbiome to modify the host niche, it is necessary to disentangle the complex dynamics of the plant pathobiome and the mechanisms by which pathogens interact or communicate with the resident host microbiota. An ultimate goal of the research in this field is to disentangle the composition and diversity patterns of plant pathobiomes, and the significance of these microbiome changes to plant health.

## Filamentous plant pathogens influence the composition of the host microbiome

Microbiome analyses by amplicon sequencing have been applied in phytopathology experiments to determine the microbiome structure (diversity, composition, and interactions) associated with healthy and diseased plants (infected or symptomatic). In such experiments, a primary objective has been to identify the microbiome members that are impacted by the presence of the pathogen and hence either increase or decrease in relative abundance (i.e., enriched or depleted, respectively). An underlying hypothesis is that infected and non-infected plants harbor different microbial communities and that these differences can reflect critical interactions between microbes.

Evidence for direct and indirect pathogen-microbiome interactions comes from a variety of different pathosystems. Root-invading pathogens, such as *Fusarium oxysporum* and *Verticillium dahliae*, modulate the microbiome composition of the rhizosphere and root endosphere where colonization occurs [24–31]. This compositional change is consistent in foliar pathogens such as *Zymoseptoria tritici*, *Albugo* spp., *Melampsora laricis-populina*, *Ustilaginoidea virens*, and *Erysiphe alphitoides*, where diseased leaves harbor different microbial communities compared to healthy leaves (including both the endosphere and phyllosphere) ([32–39]. The evaluation of local changes is relevant, as that is the place where direct microbial interaction might happen. Furthermore, changes in microbiome composition can also be detected in other plant tissues distal from the infection site, for example, the stem epidermis of chili plants infected with *F. oxysporum* [25], wheat leaves distal to the infection site of *Z. tritici* [40], and roots of plants infected with leaf pathogens such as *Magnaporthe oryzae*, *Podosphaera aphanis*, and *Blumeria graminis* [41–44]. Such systemic effects on microbiome composition are less well understood but can involve altered hormone signaling and, notably, immune-related signaling, as demonstrated in both susceptible and resistant wheat plants infected with the hemibiotrophic fungal pathogen *Z. tritici* [40].

An intriguing question is to what extent filamentous pathogens increase or decrease microbial community diversity in their host. A reduction in microbial alpha diversity is the most common pattern reported when filamentous pathogens invade plant tissues (Table S1). Particularly, plants infected with *F. oxysporum* (soybean, *A. thaliana*, banana, chili pepper), *Fusarium proliferatum* (bamboo), and *Fusarium solani* (*Hibiscus*) showed lower microbial diversity in the rhizosphere or roots in comparison with the healthy or non-inoculated plants [24–26, 28, 30, 31]. This pattern suggests that during pathogen invasion the microbiome changes can be a consequence of a loss of microbial taxa (richness), due to changes in their proportions in the host’s tissues (evenness) or both. However, in other examples, microbial diversity increases during pathogen infection [43, 45, 46] or is not changed at all [27, 29], highlighting that the impact of filamentous pathogens on plant microbiome composition is dependent on a multitude of factors, including the environmental context, physiological changes in the plant, microbiome composition, and infection development of the pathogen.

## The role of pathogen-induced immune responses on microbiome composition

As mentioned above, filamentous plant pathogens trigger and manipulate the plant’s immune response and metabolism. Disentangling the relationship of the resulting pathobiome composition, particularly the reduction of diversity or compositional changes, to the altered immune “state” of the plant versus direct interactions with the pathogen, remains a challenge. Methods to measure and characterize the immune and metabolic responses are therefore key for deciphering the importance of individual factors. For example, in the *A. thaliana*-*Hyaloperonospora arabidopsidis* pathosystem, Berendsen and colleagues [47] compared the microbiome response during pathogen infection to the response induced solely with immune activators (elicitor molecules). In this way, they were able to demonstrate that the leaf pathogen induces a salicylic acid-dependent defense response, and this particular signaling mechanism correlates with an increase in the abundance of co-occurring beneficial bacteria [47]. Plants can recruit beneficial microorganisms via metabolites (review in [48]); however, the specific recruiting mechanisms during pathogen infection remains to be explored.

Experimental designs that include resistant plant genotypes have demonstrated that microbiome diversity can be severely reduced even when pathogen infection is blocked during early hyphal penetration or when infection only causes mild symptoms. Evidence comes from experiments with fungal pathogens such as *Z. tritic*i in wheat leaves [40], *V. dahliae* in the roots of cotton [49], and *M. phaseolina* in the rhizosphere of strawberries [50]. Systemic immune responses induced by the pathogen can involve the upregulation of antimicrobial compounds with a broad impact on all other microorganisms that are colonizing the plant. For example, when the resistant wheat cultivar, Chinese Spring, is inoculated with spores of *Z. tritici*, the production of plant defense metabolites, such as benzoxazinoids and phenylpropanoids, is strongly induced [40]. Some of these compounds were experimentally shown to reduce the growth of some members of the wheat microbiome which may explain overall changes in microbiome composition in Chinese Spring [40].

## Alterations in microbiome composition can reflect plant-mediated selection for protective bacteria

Detailed analyses of community composition in diseased and healthy plants have allowed the identification of certain microbial taxa that correlate with pathogen resistance, i.e., microbial taxa that are enriched in resistant plants during pathogen infection. Such enriched microorganisms may reflect a particular selection of these taxa by the plant to confer protection against the invading pathogen. To experimentally validate the relevance of disease-resistance microorganisms, researchers rely on synthetic communities that are reduced consortia of microorganisms and intended to represent a particular microbial assembly or function [51]. Synthetic communities, which have notably been developed for and applied in *Arabidopsis*, require the extensive sampling, cultivation, and characterization of microbial members of a given plant [4].

Synthetic communities can be composed of microorganisms enriched in plants challenged with the pathogen. For example, a synthetic community composed of 13 bacteria species enriched in the roots of *F. oxysporum*-infected *Astragalus mongholicus* was found to confer protection against rot disease while a synthetic community of 13 depleted and randomly selected bacteria species did not [27]. These results support the selective enrichment of beneficial bacteria by the plant. Furthermore, Carrión and colleagues (2018) found that enriched *Chitinophaga* and *Flavobacterium* from sugar beets infected with *Rhizoctonia solani* reduce pathogen infection via enzymes and secondary metabolites, and they validated that the enrichment and antifungal activity occurred directly in response to pathogen infection [52]. Intriguingly, not only enriched bacteria but enriched fungi (*Penicillium*, *Trichoderma*, and *Gliocladiopsis*) in tea plants infected with *Pseudopestalotiopsis camelliae-sinensis* reduced disease severity [53] pointing to the importance of also studying the functional relevance of endophytic fungi.

## Inference of microbial interactions based on network analysis of plant pathobiomes

Network analysis is used to predict the putative interactions between microbial members, including antagonism when there is a negative correlation or co-existence, facilitation, and mutualisms when the correlation is positive [54]. Such predictive analyses can serve as a starting point to isolate and select candidate microbes with antagonizing effects (i.e., pathogen inhibiting). For example, in *F. oxysporum*-infected *A. mongholicus* plants, the abundance of enriched and protective bacteria was found to correlate negatively with pathogen colonization [27]. Likewise, the functional relevance of individual microbiome members, in the context of community composition, can be inferred from amplicon data using network analyses. Here, the number of “correlations” of a given microbe with other microbes serves as a measure to compute the “centrality value” of each microbial taxa and thereby assess the overall importance of the community composition. Agler and colleagues [32] successfully applied the concept of “microbial hub species” (highly connected and central species) to plant microbiome analyses and demonstrated the variability in hub species abundance in natural populations of Arabidopsis. From this study, it was found that hub species such as biotrophic pathogens belonging to the genus *Albugo* are the main drivers of microbiome community structure, particularly by reducing the alpha diversity and homogenizing the variability of the leaf microbiome [32]. A recent meta-analysis showed that the species *Albugo candida* is highly correlated, mostly negatively, with members of the leaf microbiota of *A. thaliana*, and that one of its secreted antimicrobial proteins inhibits a microbial species whose absence changes the microbiome composition and facilitates the invasion of pathogenic bacteria [55]. Since the absence of different microorganisms can have a huge impact on community structure [56], network analysis allows us to evaluate the community-level changes that pathogens induce and formulate hypotheses about the functional relevance of different members of the plant microbiome.

The robustness of microbiome networks to pathogen-induced changes can be inferred by different network metrics, particularly the number of inter- and intra-kingdom connections (positive and negative), and the number of members is often summarized as network complexity. Interestingly, the pathobiome in resistant and susceptible plants challenged with plant pathogens often has more complex bacterial or fungal networks than non-infected or non-inoculated plants [25, 26, 46, 49, 50, 57, 58]. It is plausible to speculate that more complex networks could contribute to pathogen resistance [26, 46]; however, the functional relevance of “microbiome complexity” and certain network structures remain to be tested.

## The challenge of characterizing the plant pathobionts

As highlighted above, most of the microbiome studies conducted in the framework of plant pathology aim to identify the difference between healthy and diseased plants, susceptible and resistant, or inoculated vs non-inoculated. Detailed analyses and comparisons of microbial diversity and abundance patterns in each condition are often the reported outcome of these studies. However, as suggested by Shade [59], from an ecological perspective, the diversity patterns by themselves are not the answer but rather a new question. Determining how and why a particular microbiome composition arises should be a main goal when investigating the plant microbiome during pathogen invasion. For example, to what extent is the reduction or increase of microbial diversity related to the immune response of the host or direct competition with the pathogen and other microbial community members?

One of the main inferences made in pathobiome studies is that certain taxa have the potential to act as beneficial bacteria or fungi. This relies on the prediction of lifestyles or functions solely based on species taxon (as inferred by single marker genes such as 16S or ITS). These predictions are often misleading, as functional diversity between strains of the same species or taxon is common (e.g., among *Arabidopsis*-associated bacteria of the genus *Streptomyces* which includes both beneficial and non-beneficial strains [60]). In addition, the databases used for functional inferences in microbiome studies, in some cases, rely on experimental data from human microbiome studies and may be irrelevant in a plant context [61]. Therefore, such functional predictions should be done with caution, and amplicon sequence research should ideally be accompanied by culture-dependent techniques and functional validation of the plant growth promotion, disease reduction, or pathogen antagonism either in vitro or in planta to associate certain taxa to these traits. Other -omic approaches such as metagenomics, transcriptomics, proteomics, and metabolomics can further help to elucidate what microbial functions might be relevant during pathogen infection [62]. These methods, however, also rely on database-derived information such as reference genomes and annotations.

## Filamentous plant pathogens have diverse mechanisms to interact with the plant microbiota

**Fig. 2:**
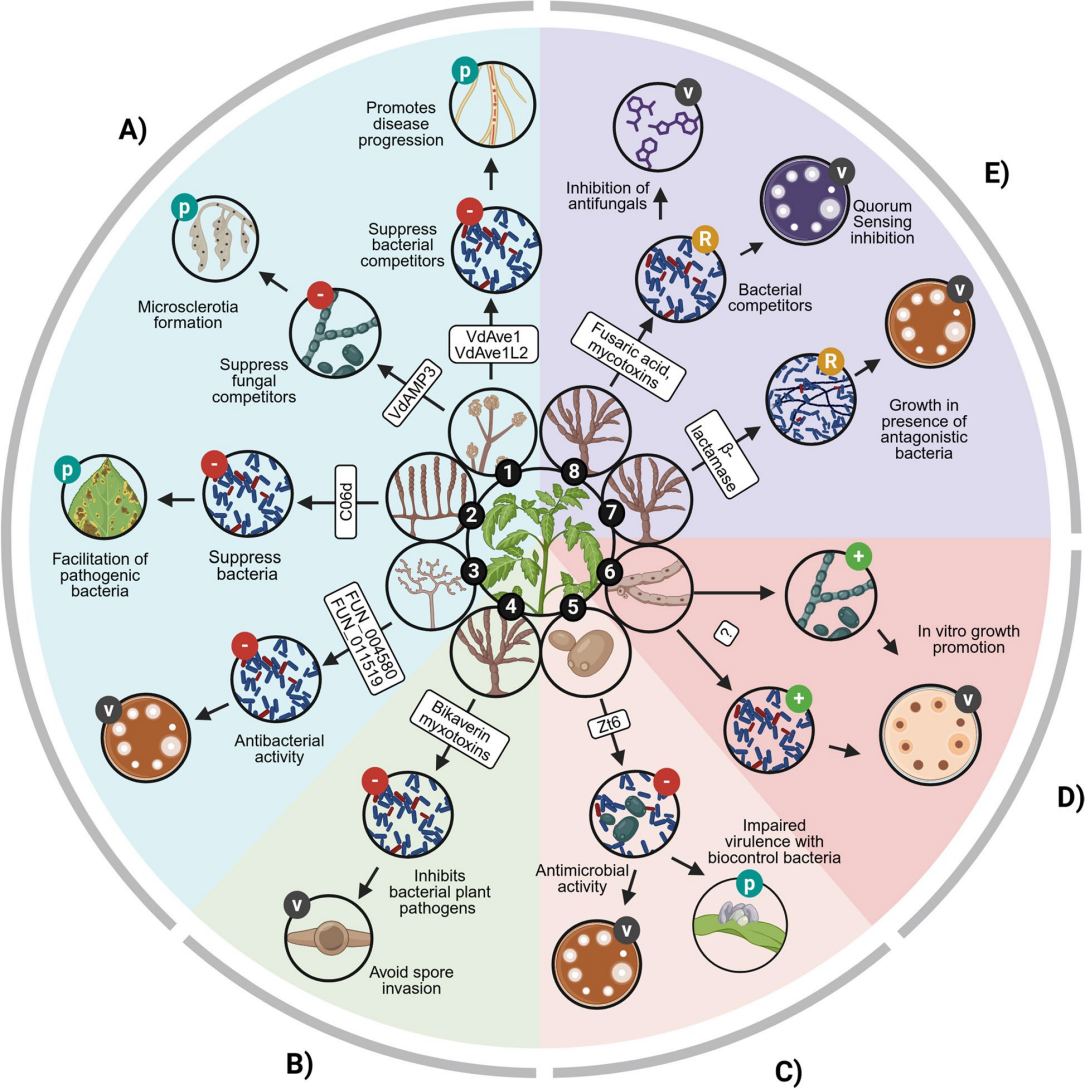
Known mechanisms whereby filamentous plant pathogens interact with the plant microbiota. Filamentous pathogens produce different effectors (white rectangles) that can interfere with microbial growth (as demonstrated in vitro) (V) and can contribute to the modification of the microbial niche and disease progression in the plant (P). Antagonistic ( −) interactions arise from the production of secreted protein effectors (A), metabolites such as mycotoxins (B), and secreted enzymes (C). Growth enhancement ( +) interactions were reported from in vitro studies, but the underlying mechanisms have not been described systematically or demonstrated in planta (D). Other non-antagonistic interactions arise from the interference of bacterial regulatory systems (R) like quorum sensing (E). Pathogens: (1) *Verticillium dahliae* [36, 37, 63], (2) *Albugo candida* [55], (3) *Rosellinia necatrix* [64], (4) *Fusarium oxysporum* [65], (5) *Zymoseptoria tritici* [66] and *Ustilago maydis* [67], (6) different fungi, such as (7) *Z. tritici* and (8) *Fusarium oxysporum* [24, 68, 69]

When a plant pathogen colonizes its host, it competes for space and resources with the native plant microbiota. Fungal pathogens can modify their microbial niche by secreting effector proteins with antimicrobial activity against different members of the plant microbiome (Fig. 2(A)). Pioneering work with the wilt pathogen *V. dahliae* showed that the fungus secretes plant natriuretic-peptide-like effectors that specifically reduce the abundance of species of *Sphingomonadales* and *Actinobacteria* which otherwise reduce pathogen invasion [36, 63]. Moreover, this pathogen secretes another effector with defensin-like folds that has antifungal activity and has a role in defending the pathogen niche during the formation of resistant structures in the senescent leaves [37]. As suggested by the authors, these findings highlight that pathogen effectors affect a specific range of taxa in a life-stage-dependent manner [20]. Interestingly, a variety of secreted antimicrobial effectors are predicted in other filamentous pathogens like *Albugo candida* and *Rosellinia necatrix* [55, 64]. It has been suggested that this mechanism is co-opted from ancient effectors in fungi by coevolving with competitive microorganisms in the soil before the emergence of plants [20, 36].

The study of effectors that mediate pathogen-microbial interaction in pathogens with host specialization allows us to develop new hypotheses concerning the mechanisms that arise from the co-evolution of pathogens, not only with their host, but also with a specific host microbiota. For example, the host-specialized *Z. tritici* secretes a phytotoxic ribonuclease during spore germination on the leaf surface and during its necrotrophic phase in wheat leaves. This toxin has necrotic activity but also antimicrobial activity against different bacteria and yeasts, and it has been hypothesized that the effector is crucial for the fungus to protect its niche during host invasion and sporulation [66]. Indeed, this toxin is also expressed in the biotrophic fungus *Ustilago maydis* during epiphytic colonization and its absence impairs virulence only in the presence of a competitive biocontrol bacteria from maize (Fig. 2 (C))[67].

Fungi produce secondary metabolites with different biological activities, some of them with antimicrobial activity [70]. Particularly, fungal pathogens produce mycotoxins that have antimicrobial activity. Bikaverin and fusaric acid are mycotoxins produced by *F. oxysporum*, both with antimicrobial activities against several pathogens and even conferring a reduction in disease severity against *Phytophthora infestans* [71]*.* Moreover, both bikaverin and beauvericin, produced by the fungal pathogens *Fusarium fujikuroi* and *B. cinerea*, have antibacterial activity against the bacterial plant pathogen *Ralstonia solanacearum* and can thus prevent bacterial invasion [65] (Fig. 2 (B)). Considering the diversity of secondary metabolites that fungi can produce, particularly the mycotoxins from fungal plant pathogens, the antagonisms via antimicrobial metabolites towards other microorganisms might be more common than previously thought.

**Fig. 3:**
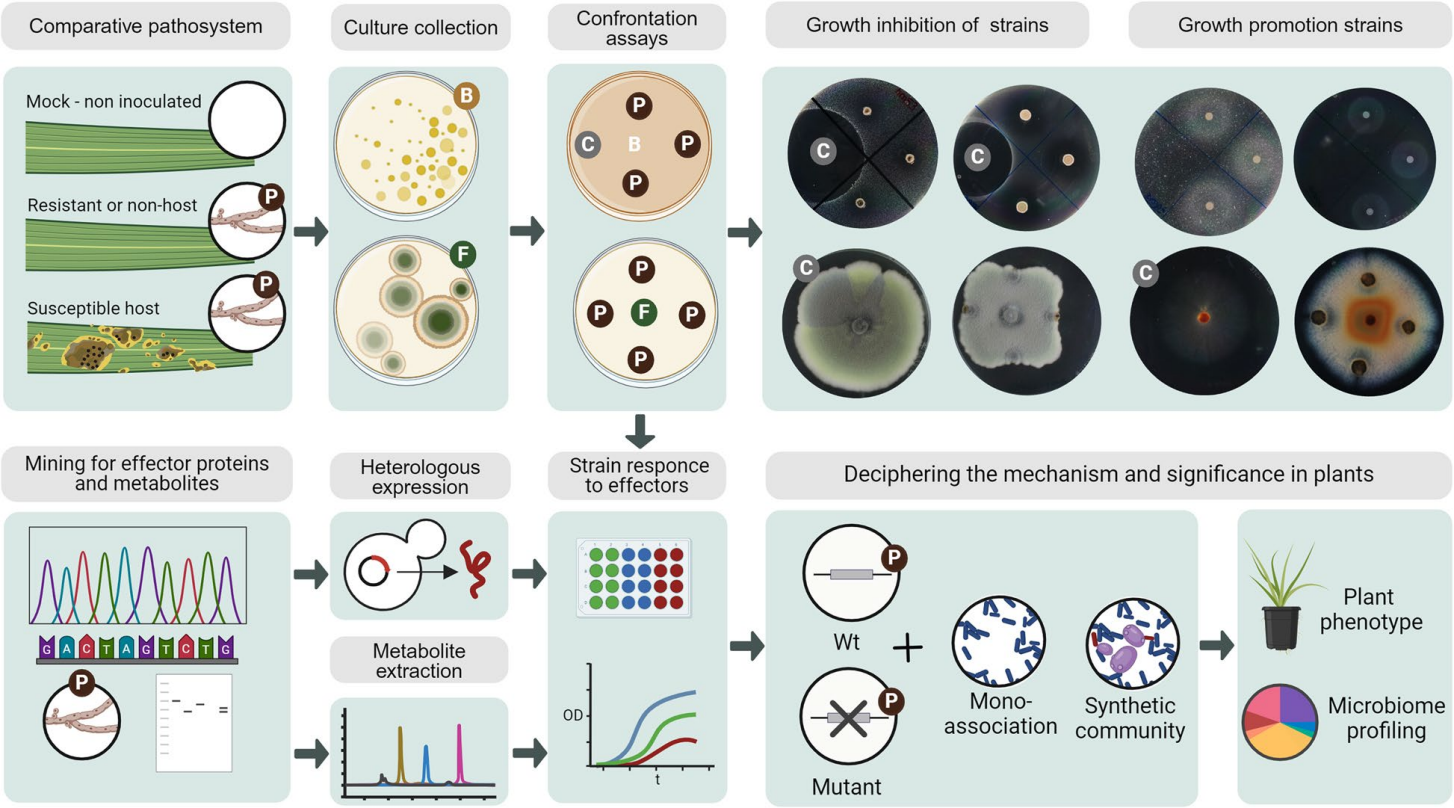
Screening of fungal and filamentous plant pathogens and the effectors that mediate their interaction with the plant microbiome. Bacteria (B) and fungi (F) can be isolated from healthy and diseased plants. In vitro confrontation assays can be performed to screen for microorganisms that might interact with the plant pathogen (P) via secreted effectors: isolated bacteria are mixed with the culture media and the pathogen is placed on top; for fungi, isolates are placed on the center of the agar plate and colonize of the pathogen are placed around it. Interaction phenotypes can vary from growth inhibition to growth promotion. Letter “C” indicates a positive control for bacterial inhibition or a growth control for filamentous fungi without the pathogen, e.g., an antimicrobial or antifungal compound. Effector proteins and metabolites can be predicted from the genomes and transcriptomes of pathogens. Their functional relevance can be characterized using molecular biology strategies: Effector proteins can be expressed and purified with heterologous expression systems, while metabolites can be separated and purified from cultures. Purified effector proteins and metabolites can then be used in in vitro assays against the microbiome culture collection. Finally, the interaction mechanism and significance can be assessed by creating deletion mutants of the effectors and mutant testing in planta together with single microbiome strains or synthetic microbial communities, where the plant phenotype and microbiome are determined.

Not all the mechanisms of microbiome manipulation involve antagonism against the microbial members. For example, *F. oxysporum* produces a beta-lactamase during in planta and in vitro interactions with the bacteria *Burkholderia ambifaria*. The secretion of this enzyme has been linked to the ability of the fungi to compete against members of the plant microbiome. However, even antagonistic strains like *Burkholderia ambifaria* increased their growth in the presence of the fungi, suggesting other mechanisms of interaction between pathogens and the microbiota present, for example, mycophagy [24]. Interaction outcomes different from antagonism and competition should be taken into consideration when studying pathogen-microbiome interactions, as the growth-stimulation of certain microbes also can confer significant changes in overall microbiome compositions. Interestingly, it has been shown that some bacteria show increased growth when challenged with secreted effector proteins in in vitro assays [36, 55]. Indeed, our culture-dependent survey of the microbiome of grasses infected with *Zymoseptoria* species showed that several plant-associated bacteria and fungi isolated from infected plant tissues increase their growth in the presence of *Zymoseptoria* when co-inoculated in in vitro assays (Flores-Nuñez et al., unpublished, Figs. 2 (D) and 3). Further experimental research should elucidate the mechanisms and ecological relevance of such growth-promoting interactions.

Quorum sensing (QS) is a cell density-dependent mechanism in bacteria and fungi that regulates many important traits. Growing evidence suggests its relevance for interkingdom communication and signaling between bacteria and fungi [72, 73]; for example, mixed biofilms of the pine endophyte *Ophiostoma pinae* with *Pseudomonas putida* are dependent on cross-signaling via QS between the partners [74]. Quorum sensing-interference has been proposed as a strategy for biocontrol of bacterial pathogens by interfering with important virulence factors [75]. However, quorum sensing interference may also be a mechanism by which pathogenic fungi inhibit the biocontrol activity of endophytic commensals [68]. Mycotoxins like fusaric acid, fumonisin, and zearalenone are QS inhibitors; in particular, fusaric acid can repress the production of QS-regulated antimicrobials such as phenizne-1-carboximide [69]. This suggests that QS inhibition potentially could be exploited by pathogens to suppress competitors or antagonists in plant tissues (Fig. 2 (E)) [68]. We propose that filamentous pathogens and microbiota communicate with and regulate each other to avoid competition or to allow their coexistence in the host.

## Final remarks

Filamentous plant pathogens manipulate the plant microbiome through direct and indirect mechanisms. The changes in the host microbiome composition come with important functional consequences such as the recruitment of beneficial microorganisms by the plant and the direct removal of microbial competitors by the pathogen. The characterization of the plant microbiota under pathogen infection has allowed us to elucidate the impact of pathogen invasion and host physiology on microbiome composition. Functional studies of crucial microorganisms rely on culture collections and the in vitro or in planta-based characterization of microbial interactions. Classical agar confrontation assays between pathogens and microbial isolates can serve as a starting point to screen for pathogens with antimicrobial activity or growth promotion activity and its microbial targets (Fig. 3). Moreover, these interactions can be contextualized in terms of the origin of the strains (healthy or diseased plants in resistant and susceptible hosts) and by the lifestyle and infection mechanism of the pathogen.

Effector-mediated modulation of microbiome composition occurs to stimulate disease progression and points to a multitude of new functions of small secreted proteins, hitherto considered mainly in the context of host immune suppression [20]. The mining of genomes to predict antimicrobial effectors and the evaluation of their expression patterns during plant colonization have proven good strategies for finding effector candidates in other filamentous pathogens. For example, the fungal pathogen *Necatrix rosea* had 26 putative antimicrobial effectors with a diverse range of structural homologs [64], while 123 antimicrobial apoplastic predicted effectors were found in *A. candida* [55]*.* The functional test of different antimicrobial domains (e.g., by heterologous expression) will shed light on the spectrum of action in the diverse microbiota of plants.

Filamentous plant pathogens compete against the host microbiota via different mechanisms, however, as summarized above, a multitude of studies have demonstrated how plant pathobiome still can harbor a diverse array of protective microorganisms. Filamentous pathogens may potentially avoid recognition of other protective microorganisms (as they do with plants) or manipulate the microbiota to avoid antagonism. Moreover, pathogen-microbiota interactions could have beneficial outcomes, such as growth promotion of other microbiome members. We speculate that such non-antagonistic interactions also occur in plants with implications on microbiome composition, function, and disease progression.

The study of pathogen-microbiota interactions has become an important topic in the field phytopathology. The functional characterization of these interactions will allow us to shed light on the role of the microbiome in disease occurrence, the evolution of filamentous plant pathogens, with the aim of delivering new insights and resources for biocontrol strategies.

### Supplementary Information

Supplementary Material 1: Table S1. Overview of studies reporting changes in the microbial alpha diversity induced by filamentous plant pathogens

## Data Availability

Not applicable.
