# Supplementary material for: The impact of filamentous plant pathogens on the host microbiota
Source: BMC Biol. 2024 Aug 15;22:175. doi: 10.1186/s12915-024-01965-3 (PMC11328434; doi:10.1186/s12915-024-01965-3)
Supplement: Supplementary file 1 — Supplementary Material 1: Table S1. Overview of studies reporting changes in the microbial alpha diversity induced by filamentous plant pathogens [file 12915_2024_1965_MOESM1_ESM.docx]

**BMC Biology. Supporting information**

**The impact of filamentous plant pathogens on the host microbiota**

**Authors:**

Victor M. Flores-Nunez^1^, Eva H. Stukenbrock^1,2^

**Affiliations:**

1. Environmental Genomics, Christian-Albrechts University, 24118 Kiel, Germany.

2. Max Planck Fellow Group Environmental Genomics, Max Planck Institute for Evolutionary Biology, 24306 Plön, Germany.

**Corresponding authors:**

[vflores-nunez@bot.uni-kiel.de](mailto:vflores-nunez@bot.uni-kiel.de) and estukenbrock@bot.uni-kiel.de

| **Table S1. Overview of studies reporting changes in the microbial alpha diversity induced by filamentous plant pathogens.** | | | | | |
| --- | --- | --- | --- | --- | --- |
| **Pathogen** | **Host** | **Plant compartment** | **Microbiome assessed** | **Alpha diversity general trend** | **DOI** |
| Ustilaginoides virens | Rice | Panicles | Bacteria, Fungi | Reduction of fungal alpha diversity, increase in bacterial alpha diversity | 10.1038/s41564-023-01379-x |
| Magnaporthe oryzae | Rice | Rhizosphere, roots | Bacteria, Fungi | Reduction of fungal alpha diversity, increase in bacterial alpha diversity | 10.1186/s12284-021-00486-9 |
| Fusarium oxysporum | Capsicum annuum | All compartments | Bacteria, Fungi | Reduction of alpha diversity | 10.1186/s40168-021-01138-2 |
| Zymoseptoria tritic | Wheat | Leaf | Bacteria, Fungi | Reduction of alpha diversity | [10.1094/PBIOMES-02-22-0008-FI](http://doi.org/10.1094/PBIOMES-02-22-0008-FI) |
| Labyrinthula zozterae | Eelgrass | Leaf | Bacteria | Reduction of alpha diversity | 10.1128/msystems.00224-22 |
| Zymoseptoria tritici | Wheat | Leaf | Bacteria | Reduction of alpha diversity | 10.1186/s40168-021-01169-9 |
| Verticillium dahliae | Tobacco | Phyllosphere | Bacteria, Fungi | Reduction of alpha diversity | 10.1073/pnas.2110968118 |
| Albugo laibachii, Albugo candida | Arabidopsis thaliana | Phyllosphere, leaf endosphere | Bacteria, Fungi | Reduction of alpha diversity | 10.1371/journal.pbio.1002352 |
| Podosphaera aphanis | Strawberry | Rhizosphere | Bacteria, Fungi | Reduction of alpha diversity | 10.1007/s00284-020-01948-x |
| Fusarium proliferatum | Bamboo | Rhizosphere | Bacteria, Fungi | Reduction of alpha diversity | [10.1016/j.indcrop.2023.116641](https://doi.org/10.1016/j.indcrop.2023.116641) |
| Fusarium oxysporum sp cubense | Banana | Rhizosphere | Bacteria, Fungi | Reduction of alpha diversity | 10.1038/s42003-023-04417-w |
| Macrophomina phaseolina | Strawberry | Rhizosphere | Bacteria, Fungi | Reduction of alpha diversity | [10.1094/PBIOMES-10-22-0069-R](https://doi.org/10.1094/PBIOMES-10-22-0069-R) |
| Fusarium oxysporum | Soybean | Rhizosphere | Bacteria, Funfgi | Reduction of alpha diversity | 10.1111/tpj.15257 |
| Verticillium dahliae | Cotton | Rhizosphere | Bacteria, Fungi | Reduction of alpha diversity | 10.3389/fmicb.2021.618169 |
| Fusarium solani | Hibiscus sabdariffa L. | Rhizosphere | Bacteria, Fungi | Reduction of alpha diversity | 10.3389/fmicb.2021.756100 |
| Phytophthora parasitica var. nicotianae | Tobacoo | Rhizosphere | Bacteria | Reduction of alpha diversity | [10.3389/fpls.2023.1152639](https://doi.org/10.3389/fpls.2023.1152639) |
| Golovinomyces orontii | Arabidopsis thaliana | Rhizosphere, root , leaf | Bacteria, Fungi | Reduction of alpha diversity | 10.1111/1462-2920.15768 |
| Plenodomus tracheiphilus | Orange | Rhizosphere, root endosphere, stem | Bacteria, Fungi | Reduction of alpha diversity | [10.3390/agronomy13030654](https://doi.org/10.3390/agronomy13030654) |
| Fusarium oxysporum | Arabidopsis thaliana | Root | Bacteria, Fungi | Reduction of alpha diversity | 10.1128/spectrum.01226-22 |
| Tilletia controversa | Wheat | Spikes | Bacteria, Fungi | Reduction of alpha diversity | [10.1128/spectrum.00390-22](https://doi.org/10.1128/spectrum.00390-22) |
| Ophiostoma novo-ulmi, | Ulmus minor | Stem endosphere | Bacteria, fungi | Reduction of alpha diversity | [10.1094/PBIOMES-04-23-0025-R](https://doi.org/10.1094/PBIOMES-04-23-0025-R) |
| Erysiphe alphitoides | Quercus ruber | Phyllosphere | Fungi | Not clear if assesed | 10.1007/s00248-016-0777-x |
| Phytophthora spp. | Pear | Rhizosphere | Bacteria | Not clear if assesed | 10.1016/j.dib.2019.104396 |
| Blumeria graminis | Barley | Rhizosphere | Bacteria | Not clear if assesed | 10.1093/femsec/fiab018 |
| Pseudopestalotiopsis camelliae-sinensis | Tea | Rhizosphere | Bacteria, Fungi | Not clear if assesed | [10.3389/fmicb.2021.774438](https://doi.org/10.3389/fmicb.2021.774438) |
| Hyaloperonospora arabidopsidis | Arabidopsis thaliana | Rhizosphere | Bacteria | Not clear if assesed | 10.1038/s41396-018-0093-1 |
| Phellinus noxius | Ficus microcarpa, Celtis sinensis, Mallotus paniculatus, Cinnamomum camphora | Rhizosphere | Bacteria, Fungi | No significant changes | 10.1002/mbo3.1115 |
| Fusarium oxysporum | Cotton | Rhizosphere | Bacteria, Fungi | No significant changes | [10.1111/1462-2920.16194](https://doi.org/10.1111/1462-2920.16194) |
| Fusarium oxysporum | Astragalus mongholicus | Rhizosphere, root | Bacteria, Fungi | No significant changes | 10.1038/s41467-020-15633-x |
| Venturia inaequalis - Podosphaera leucotricha | Apple | Rhizosphere, root | Bacteria, Fungi | No significant changes | 10.1186/s40793-023-00502-z |
| Verticillium dahliae | Cotton and Tomato | Rhizosphere, roots | Bacteria | No significant changes | 10.1038/s41477-020-00799-5 |
| Verticillium dahliae | Cotton | Root endosphere | Bacteria | No significant changes | 10.3389/fmicb.2022.906732 |
| Rhizoctonia solani | Sugar beet | Root endosphere | Bacteria | No significant changes | 10.1126/science.aaw9285 |
| Magnaporthe oryzae | Rice | Soil, rhizosphere, root, leaf | Bacteria, Fungi | No significant changes | 10.3389/fmicb.2022.949152 |
| Melampsora larici-populina | Poplar | Phyllosphere | Bacteria, Fungi | Increase of alpha diversity | 10.3390/jof8050523 |
| Fusarium oxysporum | Bean | Rhizosphere | Bacteria | Increase of alpha diversity | 10.1186/s40793-023-00524-7 |
| Botrytis cinerea | Strawberry | Rhizosphere | Bacteria | Increase of alpha diversity | [10.3389/fmicb.2016.02062](https://doi.org/10.3389/fmicb.2016.02062) |
